# Removal of contaminants of emerging concern by *Wolffia arrhiza* and *Lemna minor* depending on the process conditions, pollutants concentration, and matrix type

**DOI:** 10.1038/s41598-024-66962-6

**Published:** 2024-07-10

**Authors:** Urszula Kotowska, Janina Piekutin, Weronika Polińska, Adam Kotowski

**Affiliations:** 1 Department of Analytical and Inorganic Chemistry, Faculty of Chemistry, University of Bialystok, Ciołkowskiego 1K Str., 15-245 Bialystok, Poland; 2 Department of Environmental Engineering Technology, Faculty of Civil Engineering and Environmental Sciences, Bialystok University of Technology, Wiejska 45E, 15-351 Bialystok, Poland; 3 Doctoral School of Exact and Natural Sciences, University of Bialystok, Ciolkowskiego 1K Str., 15-245 Bialystok, Poland; 4 Department of Automatic Control and Robotics, Faculty of Electrical Engineering, Bialystok University of Technology, Wiejska 45D, 15-351 Bialystok, Poland

**Keywords:** Hormones, Bisphenol A, DEET, Phytoremediation, Removal mechanisms, Wastewater, Landfill leachates, Environmental sciences, Chemistry

## Abstract

Research was carried out on the removal of a group of six contaminants of emerging concern: bisphenol A, *N*,*N*-diethyl-*m*-toluamide, diethylstilbestrol, triclosan, estrone and estradiol from the water matrix during contact with small floating macrophytes *Wolffia arrhiza* and *Lemna minor*. The optimal conditions for the process, such as pH, light exposure per day, and plant mass, were determined using the design of experiments chemometric approach based on central composite design. Experiments conducted under the designated optimal conditions showed that after 7 days, the removal efficiency equals 88–98% in the case of *W. arrhiza* and 87–97% in the case of *L. minor*, while after 14 days of the experiment, these values are 93–99.6% and 89–98%, respectively. The primary mechanism responsible for removing CECs is the plant uptake, with the mean uptake rate constant equal to 0.299 day^−1^ and 0.277 day^−1^ for *W. arrhiza* and *L. minor*, respectively. Experiments conducted using municipal wastewater as a sample matrix showed that the treatment efficiency remains high (the average values 84% and 75%; in the case of raw wastewater, 93% and 89%, and in the case of treated wastewater, for *W. arrhiza* and *L. minor*, respectively). Landfill leachate significantly reduces plants' ability to remove pollutants (the average removal efficiency equals 59% and 56%, for *W. arrhiza* and *L. minor*, respectively).

## Introduction

Contaminants of emerging concern (CECs), including pharmaceuticals, personal care products (PPCPs), and chemicals used in the plastics industry, are increasingly being detected in water environments^1^. CECs are constantly introduced into the environment due to human activity. They are detected in concentrations ranging from nanograms per liter (sometimes picograms per liter) to micrograms per liter^2^. Nonsteroidal anti-inflammatory drugs, hormones, antioxidants, plasticisers, sunscreens, and repellents (including *N*,*N*-diethyl-*m*-toluamide (DEET), which is one of the xenobiotics most frequently detected in water) are CECs commonly detected in natural waters, especially surface waters, but also groundwater. Some compounds from the CECs group in the environment pose a potential risk to ecosystems, particularly related to their effects on the hormonal balance of organisms^3^. Estrogens, such as estrone (E1) and estradiol (E2), are endocrine disrupting compounds (EDCs) of natural origin, entering the environment mainly with runoff from municipal wastewater treatment plants (WWTPs) and farms, containing urine and feces of humans and animals. Municipal and industrial sewage, landfill leachate, and agriculture are sources of artificial EDCs, which include substances used in industry (e.g. bisphenol A (BPA)), personal hygiene products (e.g. triclosan (TRC)) and synthetic hormones (e.g. diethylstilbestrol (DES))^4,5^. The content of CECs in polluted waters varies greatly and depends on the type of compound, local conditions related to the lifestyle of the population and the method of dealing with wastewater and leachate. The concentrations of DEET, BPA, TRC, E1, E2 and DES reach the values of 6.9, 12.06, 23.9, 0.17, 0.05 and 0.02 µg/L, respectively, in the case of raw wastewater and 15.8, 10.8, 6.9, 0.08, 0.01 and 0.02 µg/L in the case of treated wastewater^6–9^. Literature data indicate that the CEC concentration in landfill leachates reaches maximum values of 431, 33,500, 42.3, 46, 1.09 and 0.88 for DEET, BPA, TRC, E1, E2 and DES, respectively^6,10,11^.

EDCs, entering organisms through the digestive, respiratory, or transdermal route, interfere with the synthesis, secretion, transport, and transformation of natural hormones or their binding to appropriate receptors. This causes several structural and functional changes in exposed organisms related to the hormonal system and the functioning of internal organs. It has been proven that exposure to EDCs, both natural, i.e. E1 and E2, and synthetic, i.e. DES, BPA, and TRC, hurts the reproduction of fish, reptiles, and birds by causing feminization of male organisms, reducing sperm quality, morphological changes in sexual organs, reduction in egg quantity and quality, and causing stress, which contributes to population reduction and deterioration of their condition^12–19^. Research has shown that TRC in the environment exerts selective pressure on exposed microorganisms, thereby altering the composition of the bacterial community^19^. DEET in aquatic organisms inhibits nutrition and reduces the population of green algae^20^. Studies on fish have proven that exposure to DEET damages androgen receptors and changes the metabolism of amino acids and carbohydrates, causes oxidative stress that damages DNA, lipids and cell membranes and triggers apoptosis^21,22^.

In humans, E1, E2, DES, BPA, and TRC have several harmful effects on the reproductive system: ovulation disorders, endometriosis, fertility problems, premature puberty, breast cancer, and uterine fibroids^23,24^. Chronic contact with these EDCs also leads to neurological disorders, cardiovascular diseases, obesity, abnormal cell proliferation, and prostate cancer^25–27^. DEET in humans can cause local (dermatitis) and systemic toxic effects. Frequent and continuous contact with DEET causes disorientation, sleeplessness, and even convulsions, unconsciousness, hypotension, and breathing problems^28^.

The growing awareness of the harmful effects of EDCs results in the introduction of standards for their content in various matrices into legislation. The European Union (EU) established guidelines for the concentrations of E2 and 17α-ethinylestradiol in water of 0.4 and 0.035 ng/L, respectively^29^. In Canada, a water quality guideline of 0.47 μg/L for TRC was established to protect wildlife aquatic organisms^30^.

One of the important ways to prevent the presence of CECs in the environment is to use effective methods of treating municipal and industrial wastewater before discharging it into natural waters or soil^31^. Common technologies used in wastewater treatment plants are based on biological, chemical, and physical methods, singly or in various combinations. In frequently applied methods based on sorption, coagulation, sedimentation, and semi-permeable membranes, contaminants are transferred unchanged from wastewater to another medium. When these methods are used, the problem of management and disposal of new solid or liquid waste arises. Biological methods lead to removing CECs by sorption, and then biodegradation occurs, i.e., the decomposition of pollutants into simpler compounds so they can be classified as technologies based on the transformation. Biological methods are usually based on microorganisms, mainly bacteria and fungi. Plants can also effectively remove CECs due to their ability to absorb and degrade harmful compounds and create a suitable environment for co-existing microorganisms, this process is called phytoremediation^32,33^.

A type of phytoremediation used in the case of polluted waters is hydrophytic treatment, in which aquatic and water-loving plants (hydrophytes) and the microorganisms living together with them are responsible for the removal of pollutants. Plants used in the phytoremediation of wastewater must be characterized by high resistance to unfavorable conditions, rapid life processes, intensive biomass growth, and the ability to absorb organic compounds from water. The most frequently used plants for this purpose are willow (*Salix viminalis L.*), deltoid poplar (*Populus deltoides*), common reed (*Phragmites australis*), broadleaf cattail (*Typha latifolia*), lake mole (*Schoenplectus lacustris*), branched coneflower (*Sparganium ramusom*), manna mullet (*Glyceria aquatica*), yellow iris (*Iris pseudoacorus*), spike weevil (*Myriophyllum spicatum*), umbel weevil (*Apium nodiflorum* L.)^34^. Rooted plants are characterized by high efficiency in removing pollutants, but their disadvantage is the long time needed to achieve the desired effect, the need to occupy a large area, and the effortfulness of cultivation. Plants floating freely on the surface of water reservoirs can also be effective in hydrophytic purification. Such plants include, among others, duckweeds *Lemna minor* L., *Lemna gibba* L., *Lemna trisulca* L., as well as the smallest vascular plant in the world, watermeal (*Wolffia arrhiza* L.), also belonging to the Lemnaceae family^35–40^. So far, little work has been devoted to possibly using the latter plant for wastewater treatment. Still, available information indicates that this plant easily acclimatizes to unfavorable external conditions, including high nitrogen concentrations, phosphorus, organic compounds, and heavy metals^41^.

Therefore, this study aimed to investigate the possibility of removing six environmentally hazardous CECs: BPA, DEET, DES, TRC, E1, and E2 from polluted waters using *W. arrhiza* and to determine the process's optimal conditions, efficiency, kinetics, and mechanism. The removal of any of these compounds by *W. arrhiza* has not been studied yet. Additionally, comparative studies were carried out using the *L. minor*, used in constructed wetlands, but its effectiveness in CECs removal is still insufficiently recognized. The novelty of this work is also a comprehensive approach to research, including the simultaneous determination of optimal process conditions, in-depth study of the mechanism, and assessment of the impact of real matrices on the efficiency of CECs removal. An important aspect of the work is to conduct tests for a mixture of compounds with different structures, in concentrations actually found in polluted waters, and to compare two plants at the same time and in the same conditions.

## Materials and methods

### Chemicals and materials

Analytical BPA, DEET, DES, TRC, E1, and E2 standards (with a purity of at least 97%) were obtained from Merck, Darmstadt, Germany. 1 mg/mL stock solutions were prepared by dissolving 10 mg of each analytical standard in 10 mL of methanol (for liquid chromatography grade, Merck, Darmstadt, Germany). The obtained solution was stored at a temperature of − 18 *°*C not longer than 2 weeks. Working solutions for removal experiments were prepared in HPLC-grade water using a MilliQ Millipore system (Bedford, MA, USA). Extraction solvent 1-undecanol was purchased in Waltham, MA, USA. Acetic anhydride (for GC derivatization ≥ 99.0%, Sigma-Aldrich, Switzerland) and potassium hydrogen phosphate (Avantor Performance Materials, Poland) were used for EDCs derivatization. Hutner’s medium used to grow *W. arrhiza* and SIS medium used to grow *L. minor* were prepared following procedures described in the literature^42,43^. All reagents used to prepare the culture media and artificial wastewater were purchased from Chempur, Poland, and Avantor Performance Materials, Poland.

*W. arrhiza* community was donated by The Faculty of Biology of the University of Bialystok (Poland). *L. minor* community was obtained from a natural population inhabiting a pond (Ożarów, central Poland). The plant collection and use were in accordance with all the relevant guidelines. Before starting the research, the plants were acclimatized for 2 weeks by placing them in an appropriate sterile culture medium and under the conditions of the experiment.

### Real matrices: wastewater and landfill leachates

Wastewater and leachate were used to determine the effect of the matrix on the removal efficiency of CECs. Raw and purified municipal wastewater samples were obtained from a municipal wastewater treatment plant (WWTP) located in northeastern Poland. The treatment process in this plant includes mechanical purification followed by biological purification through activated sludge, and no tertiary treatment is conducted. The WWTP receives wastewater from a population of 300,000. Its daily capacity equals 100,000 m^3^/day, and real processing is approximately 70,000 m^3^/day. Treatment efficiency meets the effluent standards required by Polish legislation for a plant of this size.

The landfill leachate was obtained from the municipal solid waste (MSW) landfill site for non-hazardous and inert waste in northeastern Poland. MSW landfill accepts and processes waste from a population of 300,000 people; its area equals 12.07 ha and a capacity of 677,091 m^3^. Some landfill fields are insulated with natural insulation (layers of sand, gravel, and clay), and others have synthetic insulation in the form of a 2 mm HDPE membrane. The leachate collected from landfill fields using special installations is stored in opened lagoons, and their excess is discharged to a WWTP by tanker trucks.

Wastewater and landfill leachates were collected into glass bottles and transported to the laboratory. All utensils and equipment used to collect samples were previously cleaned using an anionic detergent and thoroughly rinsed with tap water, followed by deionized water. Collected wastewater and leachates were characterized by determining the values of pollution indicators: electrolytic conductivity (EC), chemical oxygen demand (COD), biochemical oxygen demand (BOD_5_), total carbon (TC), total nitrogen (TN), and total phosphorus (TP) content. Determinations were conducted under the American Public Health Association (APHA) and American Water Works Association (AWWA) standards^44^. The values of contamination indicators characterizing raw and treated wastewater and landfill leachates used in the experiments as culture media are included in Supplementary Table S1.

### Optimization of the purification process

Optimization was performed to find the most efficient conditions for compounds removing during contact with plants. The experiments were carried out for three compounds with different structures: TRC, DES, and E2. The concentrations of each compound were 500 µg/L, Hutner's medium was used as the culture medium, and the plant used for phytoremediation was *W. arrhiza*. Design of experiments (DoE) chemometric approach based on central composite design (CCD) was applied^35,36,45,46^. Effect of three independent parameters: pH (first independent variable, x_1_), time of exposure to light per day (second independent variable, x_2_), and mass of plant per 100 ml of purified solution (third independent variable, x_3_) on the CECs removal efficiency (RE%) has been examined. Each of the examined parameters was tested on five levels.

The statistical analysis was performed according to response surface methodology (RSM) using Statistica 13.3 (Tibco Software Inc.). The number of experiments (N) in a chosen five-level plan was established based on the number of optimized variables (*k*) and the number of central points (*n*_*CP*_) and calculated from the equation:

1 $$N={2}^{k}+2k+{n}_{CP}$$

Since six central points were used, the total number of experiments was 20. The results of the conducted experiments were used to develop the second-order polynomial regression model explaining the relationship between operating variables and the response. The model used had the following form:

2 $$\begin{aligned} RE\% & = A_{0} + A_{1} x_{1} + A_{2} x_{2} + A_{3} x_{3} + A_{{11}} x_{1}^{2} + A_{{22}} x_{2}^{2} \\ & \quad + A_{{33}} x_{3}^{2} + A_{{12}} x_{1} x_{2} + A_{{13}} x_{1} x_{3} + A_{{23}} x_{2} x_{3} \\ \end{aligned}$$

where A_0_ is the constant, A_1_, A_2_, A_3_ are linear effects, A_11_, A_22_, A_33_ are the quadratic effects and A_12_, A_13_, A_23_ are interaction effects between the input factors x_1_, x_2_ and x_3_, respectively. The analysis of variance (ANOVA) has been done, and a *p*-value lower than 0.05 was considered significant in three-dimensional surface response analysis. The range of optimized parameter values was planned based on preliminary experiments, and the exact values were selected based on the coded values imposed by the model. Coded variables in the five-level CCD model are as follows: − α; − 1; 0; 1; α. Star (or axial) point α could be estimated based on the equation: $\alpha ={{(2}^{k})}^{\frac{1}{4,}}$ and for used orthogonal CCD of three independent variables, it equals 1.68^47^. Coded and real values of optimized parameters are listed in Table 1.

**Table 1:** Coded and real values of independent variables used in the CCD model.

Variables	Levels				
	− α	− 1	0	1	+ α
x_1_: pH	5	5.8	7	8.2	9
x_2_: light exposure per day (h)	10	11.2	13	14.8	16
x_3_: amount of plant per 100 mL of solution (g)	0.5	0.9	1.5	2.1	2.5

All cultivations were carried out for 7 days. After this time, culture samples were taken and processed in the way described in "Extraction, detection, and quantification of CECs in culture media" section. The GC–MS analysis was done to determine concentrations and *RE%* values for individual analytes. Each experiment was performed in triplicate.

### Removal experiments

Experiments were conducted with the mixture of all six EDCs, with the concentrations of each studied compound equal 100 µg/L or 500 µg/L. The experimental solutions were prepared by adding the appropriate amount of the CECs stock solution to the culture medium, municipal wastewater, or landfill leachate. 100 mL solutions were used when culture was carried out using culture media; in the case of wastewater and effluents, the volume of the matrix was 2 L. Experiments aimed at determining the amount of CECs accumulated in plant tissues were conducted using 5 g of *L. minor* or *W. arrhiza* per 250 mL of culture medium with the tested compounds. All dishes used in plant breeding were made of glass and were carefully washed and sterilized (baked in an oven at 120 °C for 2 h) before experiments. An appropriate portion of plants was rinsed three times with distilled water and, after thoroughly draining, placed in the sterile dish with growing solution (Hutner’s medium, SIS medium, raw wastewater, treated wastewater, or landfill leachate) enriched with CECs. The dish was covered by transparent foil (without touching the solution) to protect the growing medium against evaporation. Each experiment was run in three identical replicates. At the same time, control experiments were carried out in which plants were placed in the growing solution without adding CECs. The experiments were conducted at 23.5 ± 0.5 °C with exposure of light 13/11 h light/darkness (fluorescent light giving a photosynthetically active radiation (PAR) intensity of 50 μmol/m^2^/s). Additional experiments under different conditions were realized to explain the fate of EDCs during their removal by floating plants and to establish the participation of abiotic and biological processes such as hydrolysis, photodegradation, sorption, and plant uptake in this phenomenon. The experiments were carried out without the plant under dark conditions, without plant with exposure to light, with 1.0 g of a dead plant with exposure to light (plants were killed through a 5-day exposure to sodium azide at a concentration of 2 g/L). All other conditions, that is, medium volume, temperature, light exposure time, photon flux, and EDC concentrations, were the same. Samples of medium solution for determination of EDC concentration changes were collected and analyzed after 1, 2, 3, 5, 7, and 14 days of cultivation. Based on determined concentrations, RE% was calculated as follows:

3 $$RE\%=\left(1-\frac{C}{{C}_{0}}\right)100\%,$$

*C*_*0*_ and C are the EDC concentrations (µg/L) at the beginning and the end of the appropriate stage of the purification process.

### Extraction, detection, and quantification of CECs in culture media

The collected samples were filtered on glass fibre pre-filters (Merck, Darmstadt, Germany) and subjected to extraction and GC–MS analysis. A previously established ultrasound-assisted emulsification microextraction (USAME) procedure was used to isolate and enrich studied compounds from a growing medium^48^. In the beginning, 0.15 g of Na_2_HPO_4_ was introduced into 10 mL test tubes to which 5 mL of culture solution, 70 μL of chloroform (extractant), and 150 μL of acetic anhydride (derivatization reagent) were then introduced. The test tubes were sealed and sonicated for 5 min. Afterward, the tubes were centrifuged for 5 min (4000 rpm), and after phase separation, an organic (lower) layer was drawn into the glass chromatographic syringe and transferred to a chromatographic vial. Such a prepared sample was introduced to the GC–MS device. HP6890D gas chromatograph with a mass spectrometric detector MSD5973 and HP7673 autosampler (Agilent Technologies, USA) were used. Helium, with a purity of 99.9999%, was used as carrier gas at a 1 mL/min flow rate. The injector temperature was 250 °C. The injected sample volume was 1 µL. The apparatus was equipped with HP-5MS (5% phenylmethylsiloxane) size 30 m length × 0.25 mm, i.e., coated with 0.25 μm film thickness and split/splitless injector worked in splitless mode. The oven temperature was programmed from 150 °C, increased at 8 °C/min, to 300 °C. The total run time was 25 min. The MS detector worked in the selected ion monitoring (SIM) mode. The electron impact source temperature was 230 °C with an electron energy of 70 eV. The quadrupole temperature was 150 °C, and the GC interface temperature was 280 °C. The calibration curve method was used to carry out the quantitative determinations. To record calibration curves, a series of aqueous working solutions were subjected to an extraction procedure, and the obtained extracts were analyzed by GC–MS. The retention times of the individual compounds, the ions selected for monitoring as well as an overview of the method's performance, including linearity, limits of detection (LOD) and quantification (LOQ), repeatability, and recovery, are listed in Supplementary Table S2. An example chromatogram of CECs extracted from culture media registered under the described conditions is shown in Supplementary Fig. S1.

### Extraction and quantification of CECs in plant tissues

Sample pretreatment was done according to the procedure taken from the literature^49^. 5 g of plant material collected after 7 days of culture on enriched with CECs culture medium was effectively filtered from the remaining liquid on a glass fiber filter and dried by passing air for 15 min. After that, the plants were homogenized with 200 µL of 1 mol/L HCl methanol solution and 100 mL of acetone for 5 min in a laboratory blender (Omni Inc Bead Ruptor Elite). The sample was then shaken by hand and placed under ultrasonication (Bandelin SONOREX DIGITEC DT 102H) for 15 min at room temperature, 35 kHz ultrasound frequency, and 230 W power. The next step was filtration on glass fiber pre-filters (Merck). The extract was evaporated to dryness in a bath at 70 °C (Heidolph Hei-VAP Precision) and dissolved in 0.5 mL of acetonitrile. Then, after adding 20 mL of HCl, it was incubated for 30 min at 80 °C. This solution was extracted with methylene chloride (10 mL) and evaporated to dryness (Heidolph Hei-VAP Precision). Then, before GC–MS analysis, the sample was derivatized by adding 50 µL BSTFA (with 1% TMCS) and 50 µL pyridine to the dry residue and then heated in a sand bath for 30 min at a temperature of 68 °C. After incubation, it was cooled to room temperature and evaporated to dryness under nitrogen flow, and the dry residue was dissolved in hexane (100 µL) and analyzed by GC–MS. CEC concentrations were determined using the single-point external calibration. For this purpose, the reference sample was prepared by adding 500 μL of a methanol solution containing a mixture of tested CECs with a concentration of 1 mg/mL each to 5 g of pure plant material (concentrations of CECs in the reference sample equal to 100 μg/g). After being left for ten minutes, the obtained mixture was homogenized with HCl and acetone and subjected to the same operations and identical GC–MS analysis as samples after cultivation on culture medium enriched with CECs. The extraction and determination procedure was carried out in triplicate for cultured and reference samples. An example chromatogram of CECs extracted from plant material registered under the described conditions is shown in Supplementary Fig. S2.

## Results and discussion

### The impact of phytoremediation conditions on the CECs removal efficiency

The efficiency of phytoremediation depends to the greatest extent on the structure and chemical properties of the pollutants removed and the dynamics and nature of the plant's metabolism. The well-being of the plant used, and thus the purification results, can also be influenced by the conditions of the process. In the case of the system using a floating plant, the pH of the culture medium, the daily light exposure time, and the amount of the plant per volume of purified water were considered important for the course of the process. The experimental design chemometric approach was applied to assess the impact of selected parameters on the treatment efficiency. Such an approach reduces the number of experiments necessary for selecting optimal conditions. It saves both time and energy, which meets the assumptions of green chemistry. The characteristics of the course of experiments and the values of removal efficiency of TRC, DES, and E2 obtained for successive sets of conditions are listed in Supplementary Table S3. Based on this data, separate mathematical models were developed to characterize the relationships between the RE% and the pH, the mass of plants, and the time of exposure to the light. The statistical analysis (ANOVA) results of regression models generated for TRC, DES, and E2 are included in Supplementary Tables S4–S6.

The ANOVA results indicate that the obtained regression models are characterized by determination coefficients (*R*^*2*^) values of 0.88, 0.76, and 0.63 for DES, TRC, and E2, respectively. These values indicate that 88% of the variations in DES removal efficiency can be explained by changes in the independent variables selected for the construction of the model, and the model does not explain 12% of the observed changes. In the case of TRC and E2, the applied model explains 76% and 63% of the differences in RE%, respectively. The adjusted *R*^*2*^ values, which equal between 0.50 and 0.77, prove the high significance of the models. The optimization process was carried out for a mixture of pollutants, which causes more errors in the models but reflects the real conditions where pollutants do not occur individually.

**Figure 1:**
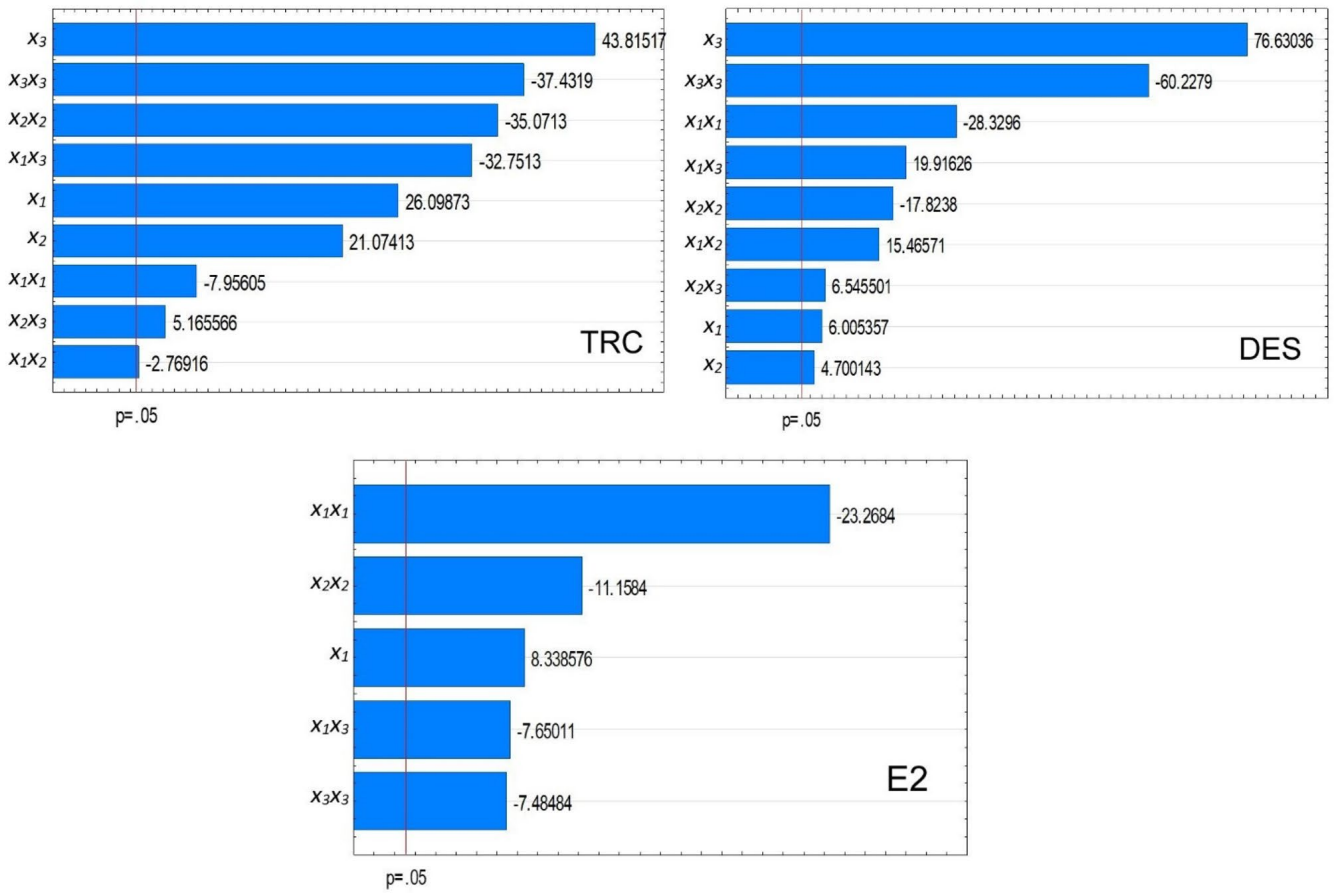
The Pareto charts for TRC, DES, and E2 (*x*_*1*_ = pH; *x*_*2*_ = light exposure per day (h); *x*_*3*_ = amount of plant (g)).

The Pareto analysis shows the importance of independent variables and interactions in the developed models^50^. Pareto charts obtained based on statistical analysis for analyzed EDCs are shown in Fig. 1.

In Fig. 1, only those parameters whose impact on the efficiency of removing is statistically significant (*p* < 0.05) were included. In the case of each CEC, all three considered parameters, i.e. lighting time, pH, and amount of plant, were significant. The obtained data indicate that the removal efficiency depends on linear values of the independent variables and quadratic values and interactions between them. In the case of DES and TRC, the greatest influence on the obtained RE% value is the weight of the plant per volume unit of the purified solution. The daily light exposure time was indicated as a factor whose significance is second in the case of TRC. pH of purified solution is the second most important factor in removing DES. The RE% of E2 depends the most on pH value, followed by light exposure time.

**Figure 2:**
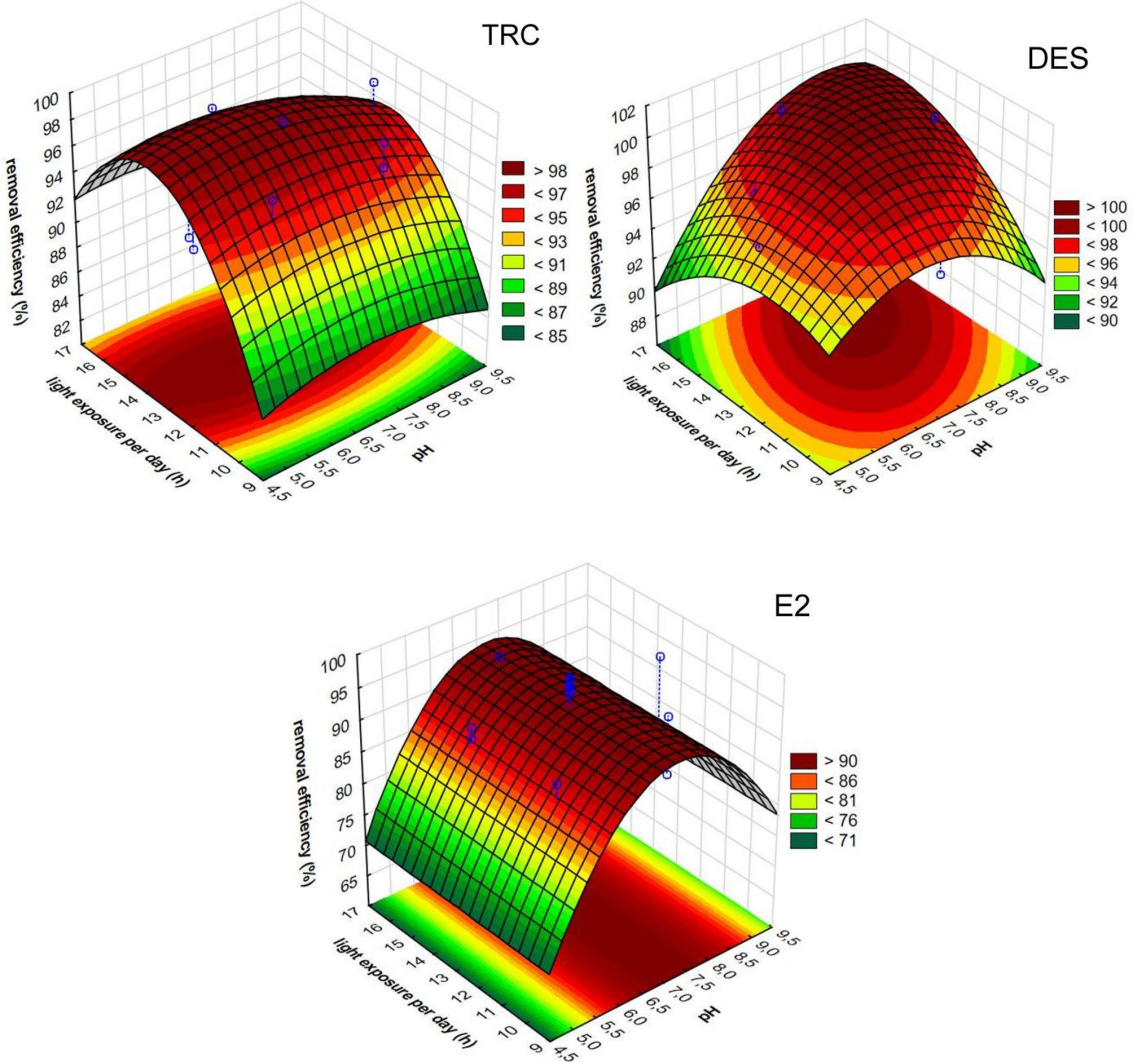
Effects of pH and light exposure time on CECs removal efficiency (mass of the plant 2 g).

Based on CCD models that only considered statistically significant data, response surface plots (RSP) were generated. The RSPs show the influence of selecting two optimized parameters on the predicted removal efficiency. Figure 2 presents the predicted RE% values from the CCD models as a function of daily light exposure and pH.

The simultaneous impact of pH and plant density, as well as the plant density and light exposure time on CECs removal rates, are included in Supplementary Figs. S3 and S4.

#### Effect of the mass of plant

Plant organisms, together with microorganisms coexisting with them, are, to the greatest extent, responsible for removing pollutants during phytoremediation. Organic micropollutants in water are adsorbed on the surface of floating plants and then collected inside the plant organism, where they undergo bioaccumulation or biodegradation. Therefore, it can be expected that the greater the mass of plants in the studied system, the greater the efficiency of the purification processes^51^. It was observed that the RE value increases as the plant density increases from 0.5 to 2.1 g per 100 ml of purified solution. Increasing plant density above these values does not increase or even decrease the observed degree of removal. This is related to the fact that density affects the growth and quality of plants^52^. Too many neighboring plants disturb reproduction, biomass accumulation, and morphology due to competition between individuals^53^. Plant density equal to 2 g per 100 mL of the purified solution was selected as the most optimal.

#### Effect of the light exposure time

The length of the light period in the diurnal cycle of 24 h is an important environmental signal for plants. They have developed mechanisms to measure the length of the photoperiod. This mechanism enables plants to synchronize developmental processes, such as the onset of flowering, with a specific time of the year. It is important in regulating responses to abiotic and biotic stresses as well as the redox state of plants^54^. During the conducted research, the influence of light exposure time in the range of 10–16 h a day on the results was examined. It turned out that extending the light exposure time improves the efficiency of CECs removal, but only to the level of 13 h a day. Further extending the duration of light action, although it promotes the intensive course of life processes, not only does it not bring benefits but also reduces the effectiveness of processes. It can be assumed that excessive exposure to light causes abiotic stress, which disturbs metabolic processes and thus reduces the removal potential of CECs by floating plants. Finally, the photoperiod equal to 13 h per day should be considered optimal from the point of view of the system's efficiency.

#### Effect of the pH of purified solution

pH affects all chemical and biological processes that occur in water. This is one of the most important factors limiting the distribution and welfare of plants in aquatic habitats. Different species thrive in different pH ranges, with the optimum value for most aquatic plants being 6.5–8. Various environmental and anthropogenic factors can contribute to lowering or upgrading the pH of water outside the optimal range. The acidifying factors are acid-generating soils and rocks, industrial and agricultural wastewater, landfill leachate, and atmospheric acid precursors. High pH is less common than low pH in natural waters, as anthropogenic sources are more often acidic than alkaline. Alkalization of water may occur under the influence of alkaline rocks and soils and runoff from the production and use of asphalt, lime, cement, and soap. The influence of pH on the results of EDC removal was assessed in the range of 5–9. In the case of the considered substances, changes in pH impacted the RE values obtained, which turned out to be the highest when the pH of the culture solution was closest to neutral. It can be concluded that such conditions are optimal for small floating plants. Both too-acidic and too-alkaline living environments negatively affect aquatic plants, causing problems with osmoregulation, tissue damage, reduced growth and reproduction^55^. Based on the tests, a pH equal to 7 was chosen as optimal. Literature data confirm the correctness of the choice. Various studies indicate that plants from the Lemnaceae family can function well at a pH of 3.5 and 10, but the optimal pH, including growth rate and protein content, is from 6.5 to 7.5^56,57^.

### Effectiveness and kinetics of CECs removal by floating plants

**Figure 3:**
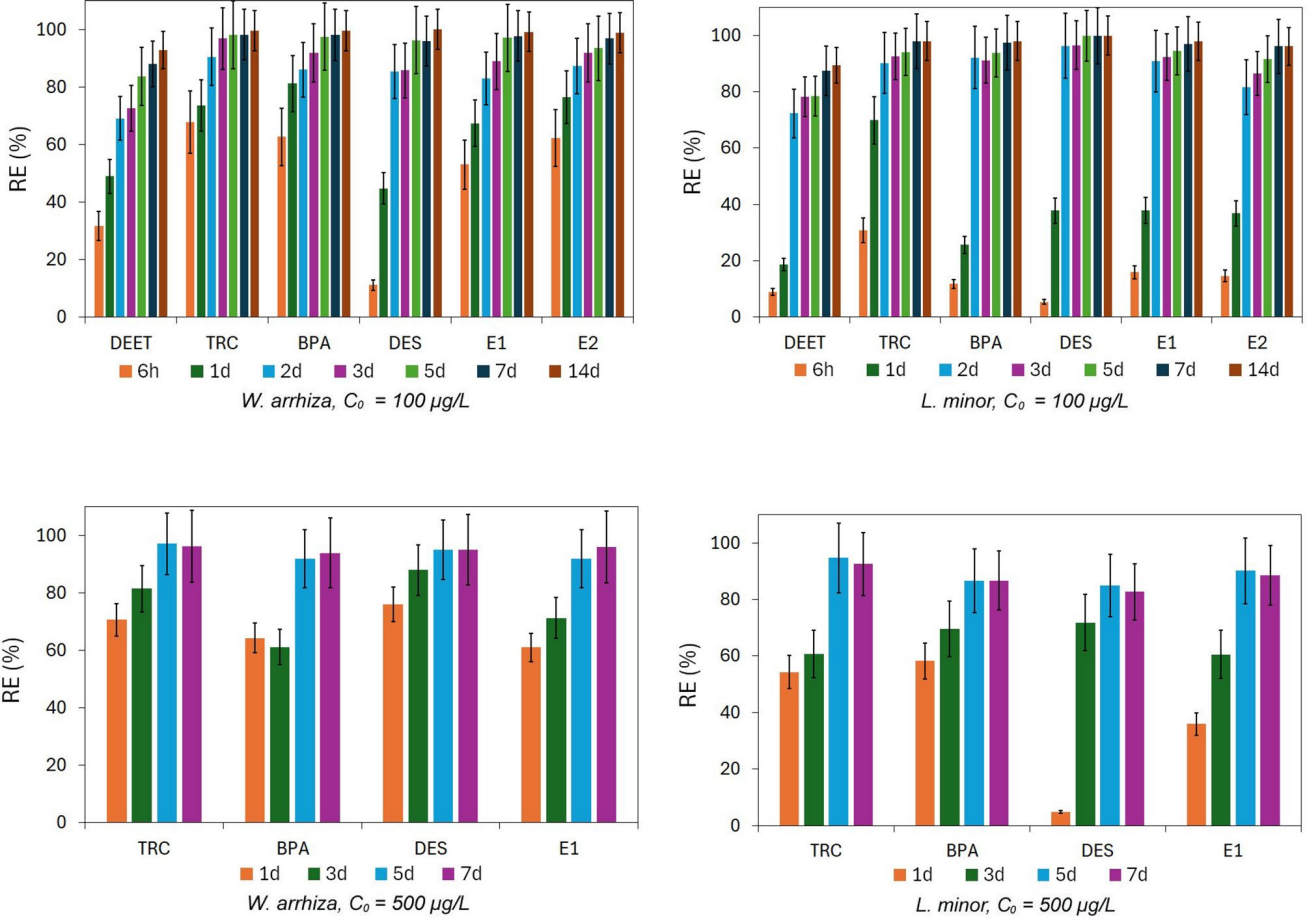
CECs removal efficiency observed during experiments with *W. arrhiza* and *L. minor*; first research cycle: *C*_*0*_ = 100 µg/L, second research cycle: *C*_*0*_ = 500 µg/L.

The course of *W. arrhi*za and *L. minor* in removing BPA, DEET, DES, TRC, E1, and E2 from the laboratory mineral culture medium was examined under the established optimal conditions. For this purpose, the floating plant cultures were set up according to the procedure in the experimental section. The initial concentration of each CEC was 100 µg/L in the first research cycle. In addition, a second test cycle was carried out in the case of TRC, BPA, DES, and E1, in which the concentrations were 500 µg/L. Current concentrations of CECs were monitored using USAEME-GC/MS method sequentially after 6 h and 1, 2, 3, 5, 7, and 14 days of *W. arrhiza* or *L. minor* cultivation in the first cycle of research and after 1, 3, 5, and 7 days of experiment in the second cycle of research. The obtained average reduction profiles are shown in Fig. 3. A gradual loss of each CEC's concentration was observed during the cultivation of both plants.

The results obtained in the first research cycle (upper graphs in Fig. 3, C0 = 100 µg/L) indicate that a quite intensive reduction of the pollutants concentrations occurred already during the first hours of the experiment, especially in vessels with *W. arrhiza*. After 6 h of contact with the medium solution with plants, the concentration of the tested compounds decreased by 11–68% in the case of *W. arrhiza* and 5–32% in the case of *L. minor*. The reduction in concentration after 1 day ranged from 45 to 81% in the system with *W. arrhiza* and from 19 to 70% in the system with *L. minor*. Seven days of contact of the solution with the plants removes 88–98% of the initial amount of CECs in the case of *W. arrhiza* and 87–97% in the case of *L. minor*. After 14 days of the experiment, these values are 93–99.6% and 89–98%, respectively. The results obtained after the first day of the second experiment cycle (lower graphs in Fig. 3, C0 = 500 µg/L) indicate a similar or even higher reduction in concentrations compared to the first cycle in the case of *W. arrhiza* (62–76%) and slightly lower in the case of *L. minor* (12–57%). After 7 days, the degree of concentration reduction is similar to the results obtained during the first experiment (94–96% *W. arrhiza*; 84–93% *L. minor*). The analysis of literature data indicates that the efficiency of removal of organic micropollutants by floating plants depends significantly on the plant species as well as the structure and properties of the removed compound^45,58,59^. Studies conducted with *W. arrhiza* have shown that the removal efficiency of phthalates by this plant ranges from 78 to over 99%^38^. In the case of benzotriazoles RE is from 23 to 100%^35^ while for benzotriazole ultraviolet stabilizers is in the range 65–92%. More data are available in the literature regarding the removal of CECs by *L. minor*. Sucralose and fluoxetine are removed by this plant by 56 and 32%, respectively^60^; cefadroxil, metronidazole, trimethoprim, and sulfamethoxazole in 100, 96, 59 and 73%, respectively^40^; diclofenac, naproxen, caffeine, ibuprofen, and clofibric acid at 99, 40, 99, 44 and 16%, respectively^61^; for benzotriazoles the RE value ranges from 20 to 81%^63^. Reinhold and coworkers studied the removal of DEET and TRC by duckweed communities consisted predominantly of *L. minor* and *Lemna punctata*^62^. The results of their research indicate that TRC is removed by plants in 97%, which is in good agreement with the results obtained by us. Similar results of TRC removal by *L. minor* are presented in the paper^61^. However, no effect of the presence of living *Lemna* plants on DEET concentration was observed compared to cultures without live plants^62^. More than 95% reduction in the concentration of E1 and E2 (initial concentration 1 µg/L) upon contact with a plants of *Lemna* species was observed after 6 days in batch experiments^63^.

By comparing the graphs ln *C* = f(*t*) and 1/*C* = f(*t*) it was determined that the rate of reduction meets the model of the pseudo-first-order kinetics. Therefore, the CECs removal rate constant (*k*) was determined based on the formula:

4 $$C_{t} = \, C_{0} e^{ - kt} ,$$

where *C*_*t*_ and *C*_*0*_ are the pollutants concentrations at time *t* and *t* = 0, respectively, (µg/L). The *k* values (day^−1^) calculated for subsequent time intervals of the conducting experiments are summarized in Supplementary Tables S7 and S8. The relevant half-life values are reported in Supplementary Table S9. In the case of experiments with *W. arrhiza*, the highest CECs removal rate is observed during the first day of contact of the plant with the solution, and the observed *k*_*1*_ values range from 0.59 to 1.67 day^−1^. The *k*_*1*_ values for experiments with *L. minor* are in the range of 0.12–1.20 day^−1^, and the highest removal rate is observed for most analytes on the second day of contact with the plant. The removal rate of CECs by *W. arrhiza* on days 0–7 ranges from 0.30 to 0.57 day^−1^ in the case of a lower concentration of compounds (100 µg/L) and from 0.40 to 0.46 day^−1^ in the case of a higher concentration (500 µg/L). In the case of *L. minor*, the observed ranges are as follows: 0.29–0.98 day^−1^ and 0.26–0.38 day^−1^ for lower and higher concentrations, respectively. The results indicate that *W. arrhiza* immediately and without problems adapts to the presence of CECs in the medium at concentrations of 100 and 500 µg/L. In the case of *L. minor*, the increase in pollutant concentrations initially reduces the intensity of CECs removal. Still, the plant adapts well to the prevailing conditions in the following days. Similar behavior of both plants was observed during previous research^21,22^.

### Removal mechanism investigation

Removal of CECs during the contact of polluted waters with plants in the applied conditions of the experiment, as well as real conditions prevailing in nature, is the sum of biological and abiotic processes. Apart from uptake by the plant followed by bioconcentration and/or biodegradation, the removal of pollutants may also be caused by hydrolysis, photolysis, and sorption (evaporation can be omitted due to the low volatility of the tested compounds)^40,64^. Additional experiments were performed under different conditions to determine the contribution of individual mechanisms to the removal of CECs. Placing the tested solutions in the dark without plants allows the evaluation of the kinetics of the hydrolysis process. When the solution is placed under lighting conditions but without plants, the removal of CECs is the sum of hydrolysis and photolysis processes. Determination of the total share of sorption, hydrolysis, and photolysis is possible when experiments are conducted with a dead plant with access to light. In studies conducted with live plants in the light, hydrolysis, photolysis, sorption, and uptake into the plant is responsible for removing CECs. The course of experiments to determine the share of individual processes in the total effect is presented in Table 2.

**Table 2:** Mechanisms responsible for the removal of CECs and the course of their determination.

Experiment	*T* (°C)	Light	Plants	Removal mechanisms
I	22 ± 2 °C	Yes	Alive	Hydrolysis, photodegradation, adsorption, plant uptake
II	22 ± 2 °C	Yes	Dead (5 days exposure for 2 mg/L NaN_3_)	Hydrolysis, photodegradation, adsorption
III	22 ± 2 °C	Yes	No	Hydrolysis, photodegradation
IV	22 ± 2 °C	No	No	Hydrolysis

The rate constants of individual processes were determined based on the following relationships^65^: *k*_*uptake*_ = *k*_*I*_–*k*_*II*_; *k*_*sorption*_ = *k*_*II*_–*k*_*III*_; *k*_*photodegradation*_ = *k*_*III*_–*k*_*IV*_; *k*_*hydrolysis*_ = *k*_*IV*_ (where *k*_*I*_, *k*_*II*_*, k*_*III*_*, k*_*IV*_ are the rate constants determined for experiments I, II, III, and IV). The detailed data of the rate constants for all obtained removal mechanisms are gathered in Table 3.

**Table 3:** The values of the plant uptake, sorption, photodegradation, and hydrolysis rate constants.

CECs	*k* (day^−1^)					
	uptake	sorption	uptake	sorption	photo-degradation	hydrolysis
	*W. arrhiza*		*L. minor*			
DEET	0.385	0.038	0.023	0.131	0.029	0.113
TRC	0.385	0.059	0.410	0.022	0.006	0.124
BPA	0.109	0.269	0.251	0.083	0.061	0.123
DES	0.753	0.060	0.559	0.254	0.005	0.169
E1	0.105	0.327	0.344	0.055	0.004	0.101
E2	0.055	0.229	0.074	0.192	0.050	0.155

Based on the averaging of the determined values, it can be concluded that the largest contribution to the removal of CECs is the plant uptake, with the mean values of *k*_*uptake*_ equal to 0.299 day^−1^ and 0.277 day^−1^ for *W. arrhiza* and *L. minor*, respectively. Interestingly, despite the similarity of both plants, the uptake of DEET by *W. arrhiza* is characterized by high intensity, while it has a small share in the removal of this compound by *L. minor*. The obtained results confirm the observations made by Reinhold and colleagues, who also did not observe a significant impact of biological processes on the efficiency of DEET removal^62^. Sorption is the dominant mechanism for removing BPA, E1, and E2 by *W. arrhiza* and DEET and E2 by *L. minor*. It should be emphasized that the processes of sorption and plant uptake are closely related because the pollutant must first be adsorbed on the surface of roots or leaves to be introduced into the plant organism^39^. The average *k*_*sorption*_ values are about half that of *k*_*uptake*_ and are 0.164 day^−1^ and 0.123 day^−1^ for *W. arrhiza* and *L. minor*, respectively. Hydrolysis's contribution in removing CECs is similar to the contribution of sorption; the average value of *k*_*hydrolysis*_ for the tested compounds is 0.131 day^−1^. Studies have shown that DEET, TRC, BPA, DES, E1, and E2 are very slightly susceptible to photodegradation; the rate constant of this process (*k*_*photodegradation*_) equals only 0.026 day^−1^.

**Figure 4:**
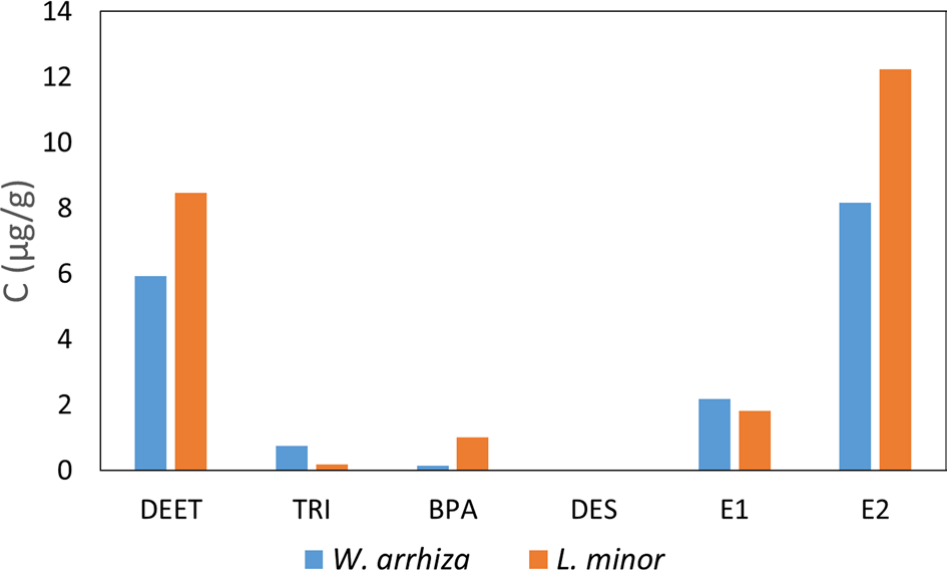
The concentration of CECs in *W. arrhiza* and *L. minor* tissues after 7-days cultivation in culture medium enriched with DEET, TRI, BPA, DES, E1, E2 (100 µg per 1 g of the plant).

As part of the mechanism assessment of CECs removal, the concentrations of the tested compounds in the plant material were determined after a 7-day contact with the enriched nutrient solution containing 100 µg of each compound per 1 g of *W. arrhiza* or *L. minor*, following the procedure described in "Extraction, detection, and quantification of CECs in culture media" section. The experiment conducted in this way allows for the assessment of what part of the tested compound that has been adsorbed and taken up by the plant remains accumulated in the plant. The results of the experiment are shown in Fig. 4.

As can be seen in Fig. 4, no DES was detected in the plants, while the content of the remaining ECs ranged from 0.15 µg/g (BPA) to 8.17 µg/g (E2) in the case of *W. arrhiza* and from 0.19 µg/g (TRI) to 12.23 µg/g (E2) in the case of *L. minor*. Based on the rate constant data listed in Table 3, it can be calculated that, under the conditions of the experiment, the total amount of individual CECs that were adsorbed and uptake by plants ranges from 58 µg/g (E2) to 82 µg/g (DES) in the case of *W. arrhiza* and from 52 µg/g (DEET) to 82 µg/g (DES) in the case of *L. minor* (because the sum of *k*_*sorption*_ and *k*_*uptake*_ constitutes from 58 to 82% and from 52 to 82% of the sum of the rate constants of all processes, for *W. arrhiza* and *L. minor*, respectively). The obtained results indicate that the plant takes up the vast majority of CECs after adsorption and that only a small part of the collected organic compounds is accumulated in their original form. Organic compounds introduced into a plant cell may be immobilized there unchanged and/or undergo enzymatic modifications, enzymatic degradation, or conjugation with other compounds, mainly glucose and glutathione^39^. The uptake of organic compounds into plants and their further fate is affected by many factors, such as chemical hydrophobicity, molecular ionization, and the tendency of the compound to undergo sorption, which precedes the introduction of the compound into the plant^65,66^. The values of the logarithm of the tested CECs partition coefficients in the *n*-octanol/water system (log *K*_*ow*_) are 2.02, 4.76, 3.32, 5.07, 3.43, and 3.94. The logarithm values of the soil organic carbon adsorption coefficient (log *K*_*oc*_) are 1.97, 4.28, 3.18, 4.68, 3.16, and 3.52 for DEET, TRC, BPA, DES, E1, and E2, respectively^67–70^. In the studied group, DES is the compound with the highest log *K*_*ow*_ and log *K*_*oc*_ values. This compound is characterized by the highest level of sorption and uptake by plants (82% of total removal), and the most intense transformations in the plant (no presence in unchanged form in the tissues of *W. arrhiza* and *L. minor*). DEET, characterized by the lowest hydrophobicity and tendency to sorption, is much less subject to both uptake by the plant and changes inside it. However, there is no strict correlation between the log *K*_*ow*_ and log *K*_*oc*_ values and the fate of CECs because other factors, such as molecular volume, spatial structure, etc., also influence the processes. Ionization has also been shown to influence the plant's uptake and modifications of organic compounds because charged molecules have a reduced ability to permeate cell membranes^71^. The values of the negative logarithm of the dissociation constants (*pK*_*a*_) of the tested compounds are 7.9, 9.6, 9.1, 10.8, and 10.7, respectively, for TRC, BPA, DES, E1, and E2 (no data for DEET)^67,68^. Under the experimental conditions (pH 7), the tested compounds are in a non-ionized form, which is beneficial for phytoremediation.

### Influence of the matrix on the course of phytoremediation

**Figure 5:**
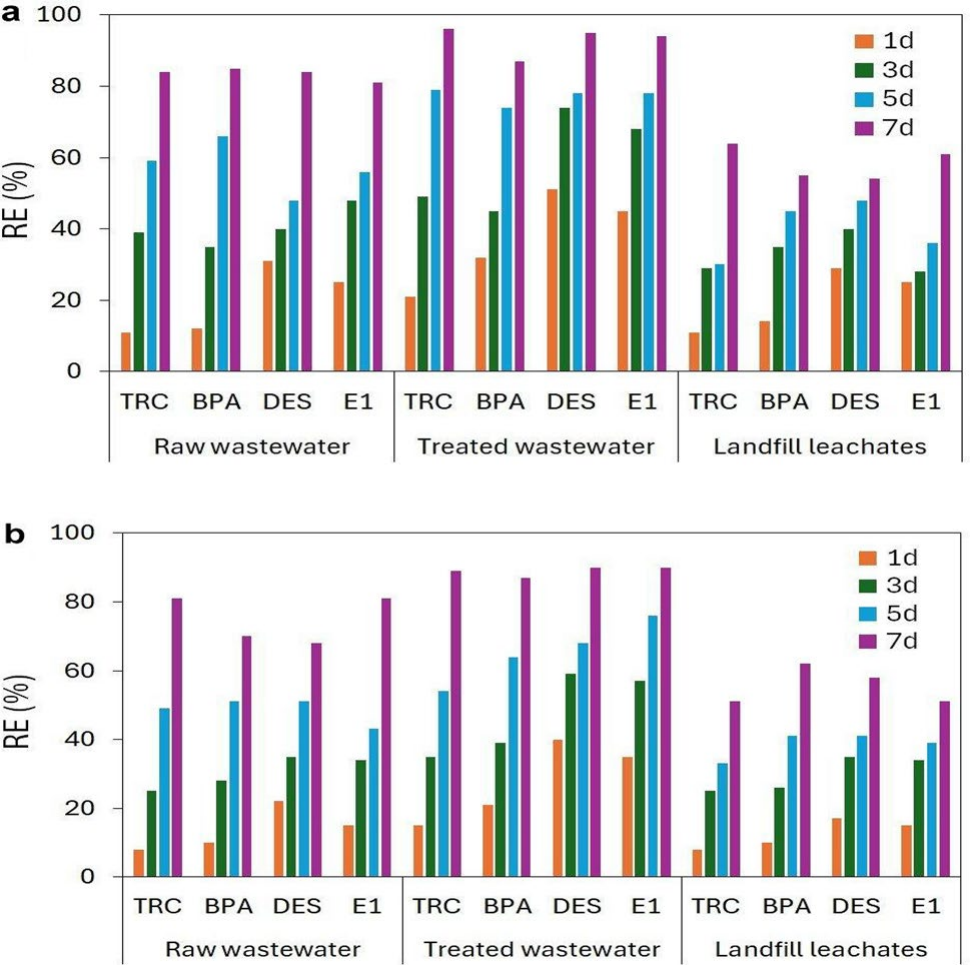
The removal efficiency of CECs registered after 1, 3, 5 and 7 days of contact of raw municipal wastewater, treated municipal wastewater and landfill leachates with *W. arrhiza* (**a**), *L. minor* (**b**).

Experiments were carried out using raw municipal wastewater, treated municipal wastewater, and raw landfill leachate enriched with a mixture of TRC, BPA, DES, and E1 at 100 µg/L each as a culture medium, maintaining optimal conditions used in previous cultures. The study lasted 7 days; the concentrations of the tested compounds were determined before the start of the experiment and after 1, 3, 5, and 7 days of contact of the plant with the solution. The CECs removal efficiency obtained using real matrices is shown in Fig. 5.

In the case of raw wastewater, the average removal efficiency of the tested compounds after 7 days of treatment was 84% and 75%; in the case of treated wastewater, 93% and 89%, and in the case of landfill leachate, 59% and 56%, for *W. arrhiza* and *L. minor*, respectively. The observed effectiveness of *W. arrhiza* and *L. minor* is similar for each matrix used, with a slight advantage for *W. arrhiza*. A worse removal efficiency by *L. minor* is visible mainly in the first days of the experiment. This may indicate a more difficult acclimatization of this plant to the presence of a highly contaminated matrix. The high efficiency of CECs removal from raw wastewater indicates the resistance of the tested plants to the presence of pollutants. Raw municipal wastewater contains certain amounts of nutrients (N and P, see Table S1, Supplementary Material) that support the growth and development of plants. Additionally, there are high concentrations of easily digestible organic compounds, including carbohydrates, fats, and proteins, which may also positively affect the functioning of plants from the Lemnaceae family, given their well-documented ability to provide mixotrophic nutrition^71^. The observed ability of floating plants to remove CECs from landfill leachates is significantly lower than in the case of municipal wastewater. Leachate is a liquid that is a toxic cocktail containing high concentrations of pollutants such as soluble organic matter, inorganic components, heavy metals, and xenobiotic organic compounds^72^. Leachates are characterized by high phytotoxicity^73^, so it can be assumed that the removal of the tested compounds during the experiment is carried out to a small extent by biological processes, mainly by sorption, hydrolysis, and photolysis.

## Conclusions

Studies have shown that floating aquatic plants *W. arrhiza* and *L. minor* effectively remove the CECs from the contaminated water. The mass of the plant, daily light exposure, and pH of the purified solution have a significant influence on the removal efficiency. The highest rate of the process is observed during the first day in the case of *W. arrhiza* and the second day in the case of *L. minor*. Increasing concentration of pollutants initially disturbs the activity of *L. minor*, but in the following days, the plant acclimatizes well to the process conditions. The mechanism that is most responsible for removing pollutants is plant uptake, followed by sorption and hydrolysis. Only a small part of the CECs taken up by plants is accumulated in their tissues unchanged, and most of them are transformed or degraded. The type of matrix affects the efficiency of pollutant removal by floating plants but it remains at a very high level in the case of treated wastewater, which means that the process can be used for post-treatment of effluents from municipal WWTP. The presence of landfill leachates has a toxic effect on plants and significantly reduces their effectiveness.

### Supplementary Information

Supplementary Information.

## Data Availability

The authors declare that the data supporting the findings of this study are available within the paper and its Supplementary Information files. Should any raw data files be needed in another format, they are available from the corresponding author upon reasonable request.
