# Supplementary material for: Removal of contaminants of emerging concern by Wolffia arrhiza and Lemna minor depending on the process conditions, pollutants concentration, and matrix type
Source: Sci Rep. 2024 Jul 10;14:15898. doi: 10.1038/s41598-024-66962-6 (PMC11237155; doi:10.1038/s41598-024-66962-6)
Supplement: Supplementary file 1 — Supplementary Information. [file 41598_2024_66962_MOESM1_ESM.docx]

Supplementary Information for

**Removal of contaminants of emerging concern by** ***Wolffia arrhiza* and *Lemna minor* depending on the process conditions, pollutants concentration, and matrix type**

Urszula Kotowska^1*^, Janina Piekutin^2^, Weronika Polińska^3^, Adam Kotowski^4^

^1^Department of Analytical and Inorganic Chemistry, Faculty of Chemistry, University of Bialystok, Ciołkowskiego 1K Str., 15-245 Bialystok, Poland; ukrajew@uwb.edu.pl

^2^Department of Environmental Engineering Technology, Faculty of Civil Engineering and Environmental Sciences, Bialystok University of Technology, Wiejska 45E, 15-351 Bialystok, Poland; [j.piekutin@pb.edu.pl](mailto:j.piekutin@pb.edu.pl)

^3^Doctoral School of Exact and Natural Sciences, University of Bialystok, Ciolkowskiego 1K Str., 15-245 Bialystok, Poland; [w.polinska@uwb.edu.pl](mailto:w.polinska@uwb.edu.pl)

^4^Department of Automatic Control and Robotics, Faculty of Electrical Engineering, Bialystok University of Technology, Wiejska 45E, 15-351 Bialystok, Poland; [a.kotowski@pb.edu.pl](mailto:a.kotowski@pb.edu.pl)

Table S1. The values of pollution indicators characterizing raw and treated wastewater and landfill leachates used in the experiments

|  | *Raw  wasteater* | *Treated wastewater* | *Landfill leachates* |
| --- | --- | --- | --- |
| Chemical oxygen demand (COD), mg/L | 315 | 31 | 2964 |
| Biological oxygen demand (BOD_5_), mg/L | 89 | 7 | 400 |
| pH | 8.29 | 7.82 | 8.69 |
| Electrolytic conductivity, mS/cm | 1,755 | 961 | 2,102 |
| Total nitrogen concentration, mg/L | 46.5 | 10.2 | 307.7 |
| Total phosphorus concentration, mg/L | 2.29 | 0.14 | 10.47 |

Table S2. The target CECs retention times, the ions selected for monitoring, and an overview of the USAEME-GC-MS concentration determination method's performance

|  | Retention time (min) | Quantification and identifi-cation ions (m/z) | Linearity range (µg/L) | *R*^2^ | LOD  (µg/L) | LOQ  (µg/L) | RSD  (%) | Recovery  (%) |
| --- | --- | --- | --- | --- | --- | --- | --- | --- |
| DEET | 5.14 | 91, 119, 190 | 0.5-500 | 0.9954 | 0.01 | 0.03 | 11.8 | 96 |
| TRC | 16.08 | 218, 288, 290 | 0.5-500 | 0.9987 | 0.01 | 0.03 | 6.4 | 109 |
| BPA | 19.08 | 213, 228, 270 | 0.5-500 | 0.9971 | 0.01 | 0.03 | 9.5 | 108 |
| DES | 21.23 | 268, 310, 352 | 0.5-500 | 0.9961 | 0.01 | 0.03 | 11.2 | 104 |
| E1 | 24.16 | 185, 270, 272 | 0.5-500 | 0.9933 | 0.02 | 0.06 | 11.6 | 93 |
| E2 | 24.40 | 43, 146, 272 | 0.5-500 | 0.9982 | 0.02 | 0.06 | 11.1 | 112 |


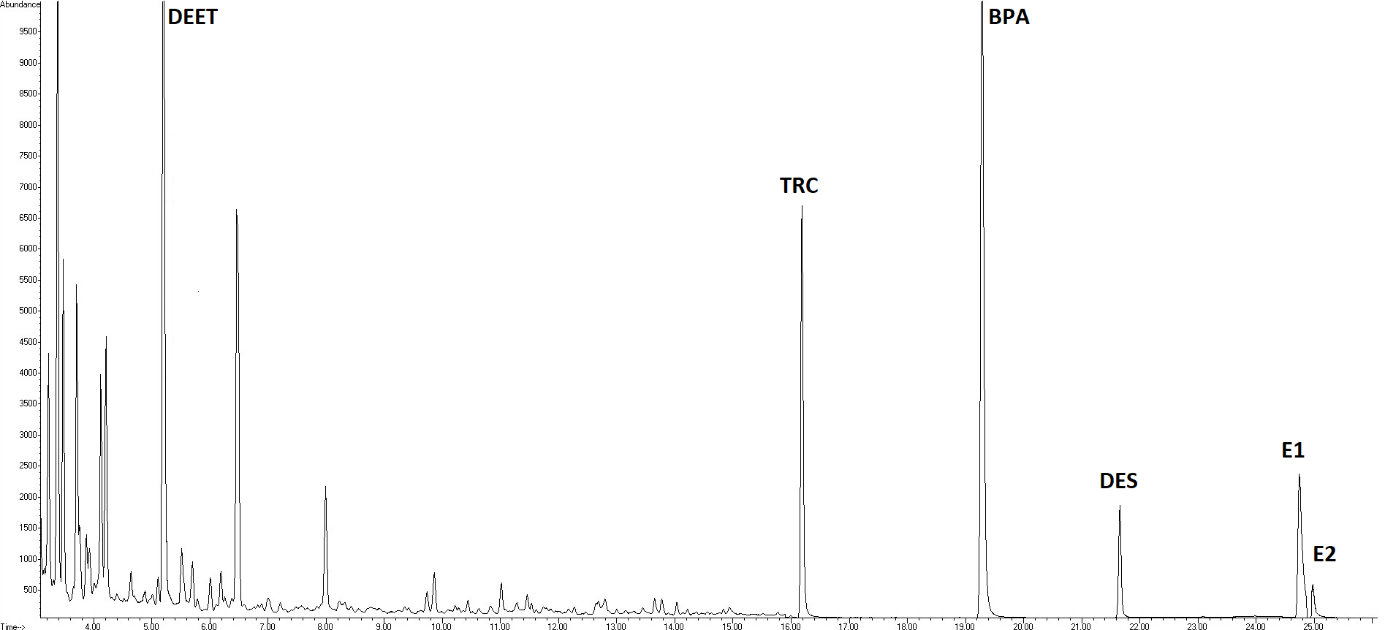


Figure S1. Chromatogram of CECs extracted from cultivation medium


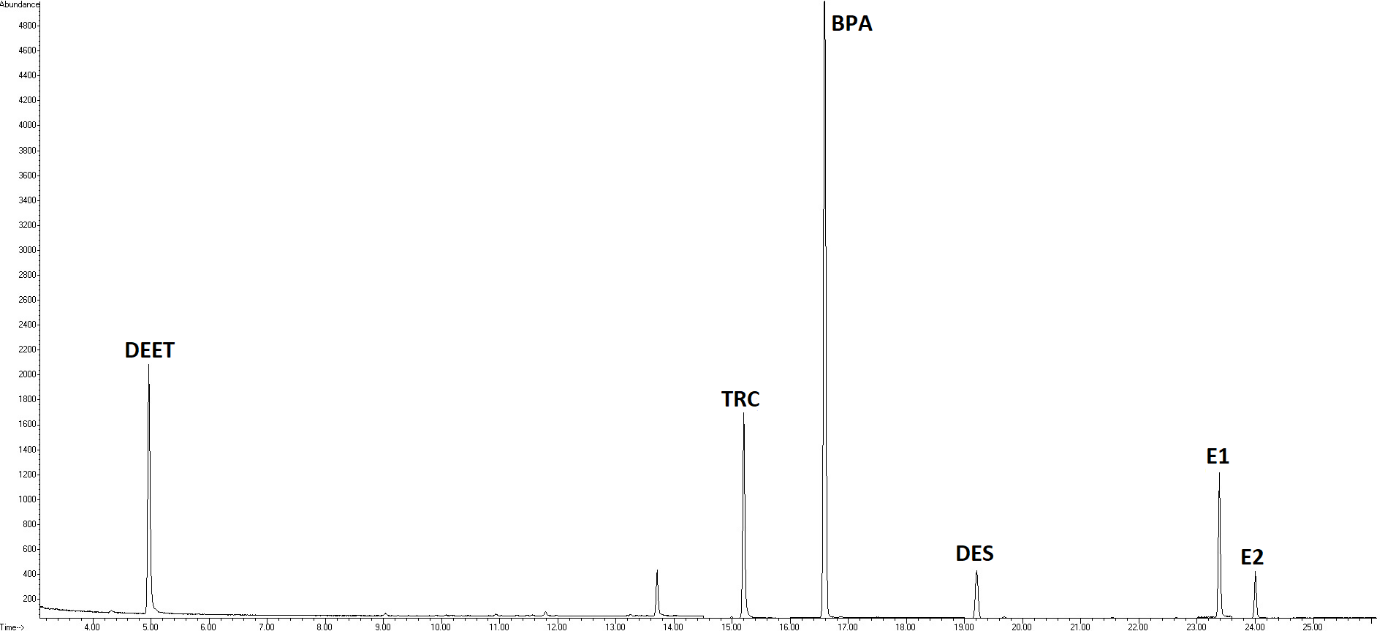


Figure S2. Chromatogram of CECs extracted from plant material

Table S3. The characteristics of the course of experiments and the values of removal efficiency of TRC, DES, and E2 obtained for successive sets of conditions

| Run | pH | Light exposure time (h) | Amount of plant (g) | Removal efficiency (%) | | |
| --- | --- | --- | --- | --- | --- | --- |
|  |  |  |  | DES | TRC | E2 |
| 1 | 8.2 | 14.8 | 2.1 | 98 | 94 | 82 |
| 2 | 8.2 | 14.8 | 0.9 | 93 | 96 | 88 |
| 3 | 8.2 | 11.2 | 2.1 | 97 | 95 | 84 |
| 4 | 8.2 | 11.2 | 0.9 | 92 | 97 | 93 |
| 5 | 5.8 | 14.8 | 2.1 | 97 | 97 | 90 |
| 6 | 5.8 | 14.8 | 0.9 | 93 | 89 | 88 |
| 7 | 5.8 | 11.2 | 2.1 | 98 | 97 | 90 |
| 8 | 5.8 | 11.2 | 0.9 | 96 | 90 | 90 |
| 9 | 9 | 13 | 1.5 | 100 | 99 | 95 |
| 10 | 5 | 13 | 1.5 | 96 | 93 | 74 |
| 11 | 7 | 16 | 1.5 | 100 | 96 | 95 |
| 12 | 7 | 10 | 1.5 | 98 | 86 | 89 |
| 13 | 7 | 13 | 2.5 | 100 | 98 | 96 |
| 14 | 7 | 13 | 0.5 | 90 | 85 | 92 |
| 15 | 7 | 13 | 1.5 | 99 | 98 | 96 |
| 16 | 7 | 13 | 1.5 | 99 | 98 | 97 |
| 17 | 7 | 13 | 1.5 | 99 | 98 | 97 |
| 18 | 7 | 13 | 1.5 | 99 | 98 | 97 |
| 19 | 7 | 13 | 1.5 | 99 | 99 | 98 |
| 20 | 7 | 13 | 1.5 | 99 | 98 | 98 |

Table S4. The ANOVA results of regression model generated for TRC

| Source of variation | Sum of squares | DF | Mean square | *F*-value | *p*-value |
| --- | --- | --- | --- | --- | --- |
| pH (Linear) | 27.8639 | 1 | 27.86394 | 681.144 | 0.000002 |
| pH (Square) | 2.5894 | 1 | 2.58940 | 63.299 | 0.000506 |
| Light (Linear) | 18.1678 | 1 | 18.16784 | 444.119 | 0.000004 |
| Light (Square) | 50.3161 | 1 | 50.31609 | 1229.994 | 0.000000 |
| Amount of plant (Linear) | 78.5331 | 1 | 78.53314 | 1919.769 | 0.000000 |
| Amount of plant (Square) | 57.3174 | 1 | 57.31743 | 1401.144 | 0.000000 |
| pH-light Interactions | 0.3137 | 1 | 0.31369 | 7.668 | 0.039404 |
| pH-amount of plant Interactions | 43.8795 | 1 | 43.87946 | 1072.648 | 0.000000 |
| Light-amount of plant Interactions | 1.0915 | 1 | 1.09154 | 26.683 | 0.003568 |
| Pure error | 0.2045 | 5 | 0.04091 |  |  |
| Total | 349.7372 | 19 |  |  |  |
| R2=0.75623 | R2 (Adjusted)=0.53684 | |  |  |  |

Table S5. The ANOVA results of regression model generated for DES

| Source of variation | Sum of squares | DF | Mean square | *F*-value | *p*-value |
| --- | --- | --- | --- | --- | --- |
| pH (Linear) | 0.4736 | 1 | 0.47361 | 36.064 | 0.001839 |
| pH (Square) | 10.5396 | 1 | 10.53961 | 802.568 | 0.000001 |
| Light (Linear) | 0.2901 | 1 | 0.29011 | 22.091 | 0.005336 |
| Light (Square) | 4.1720 | 1 | 4.17197 | 317.686 | 0.000010 |
| Amount of plant (Linear) | 77.1160 | 1 | 77.11599 | 5872.211 | 0.000000 |
| Amount of plant (Square) | 47.6363 | 1 | 47.63627 | 3627.396 | 0.000000 |
| pH-light Interactions | 3.1411 | 1 | 3.14111 | 239.188 | 0.000021 |
| pH-amount of plant Interactions | 5.2090 | 1 | 5.20905 | 396.657 | 0.000006 |
| Light-amount of plant Interactions | 0.5626 | 1 | 0.56264 | 42.844 | 0.001246 |
| Pure error | 0.0657 | 5 | 0.01313 |  |  |
| Total | 162.3961 | 14 |  |  |  |
| R2=0.87931 | R2 (Adjusted)=0.77069 | |  |  |  |

Table S6. The ANOVA results of regression model generated for E2

| Source of variation | Sum of squares | DF | Mean square | *F*-value | *p*-value |
| --- | --- | --- | --- | --- | --- |
| pH (Linear) | 40.4872 | 1 | 40.4872 | 69.5319 | 0.000406 |
| pH (Square) | 315.2594 | 1 | 315.2594 | 541.4196 | 0.000003 |
| Light (Square) | 72.5001 | 1 | 72.5001 | 124.5101 | 0.000101 |
| Amount of plant (Square) | 32.6211 | 1 | 32.6211 | 56.0228 | 0.000673 |
| pH-amount of plant Interactions | 34.0776 | 1 | 34.0776 | 58.5242 | 0.000608 |
| Pure error | 2.9114 | 5 | 0.5823 |  |  |
| Total | 712.9060 | 19 |  |  |  |
| R2=0.6318 | R2 (Adjusted)=0.5003 | |  |  |  |

| 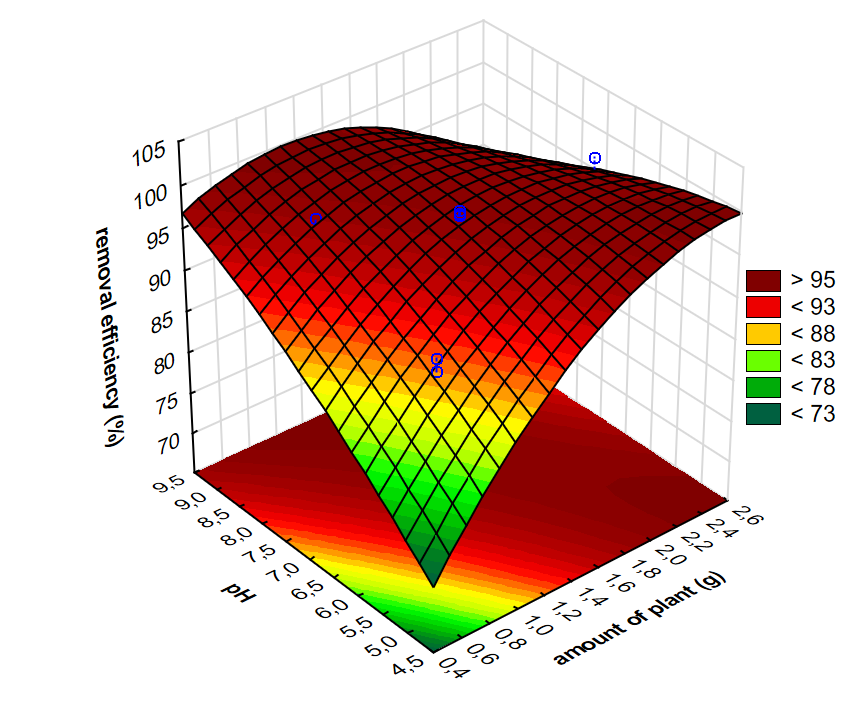 |
| --- |
| 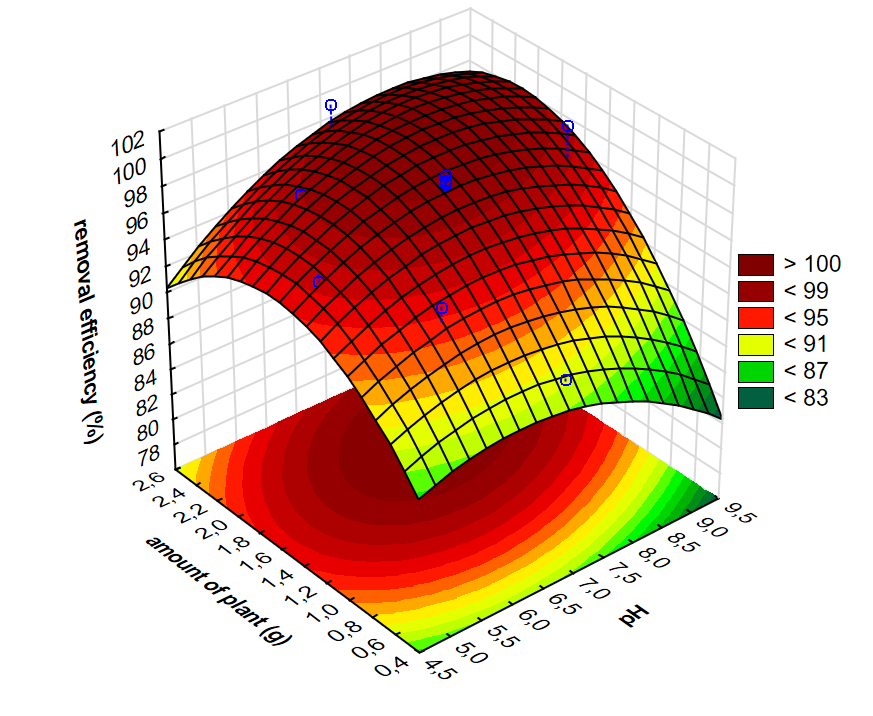 |
| 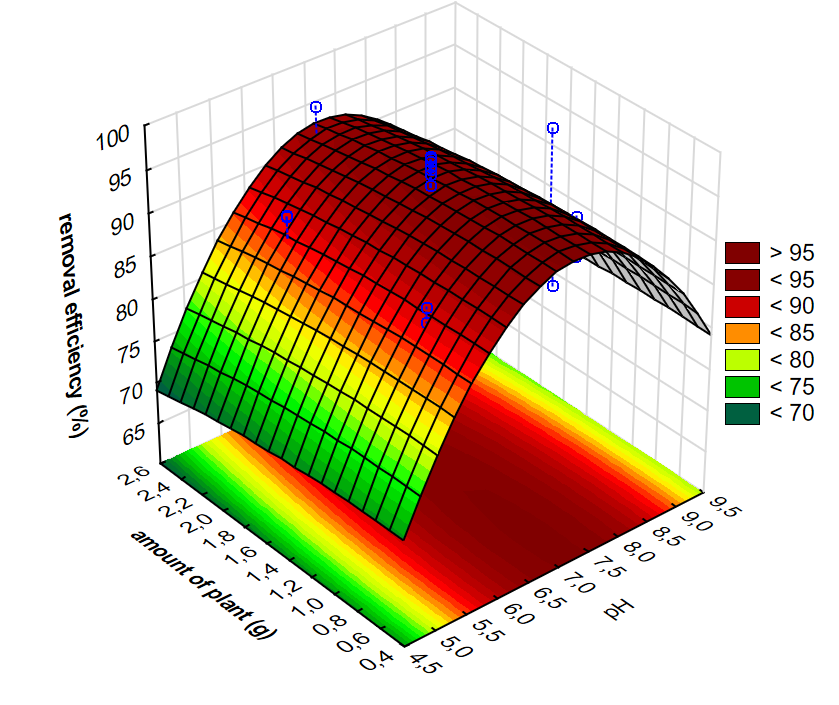 |

Figure S3. Effects of pH and mass of plant on EDCs removal rate by *W*. *arrhiza*

| 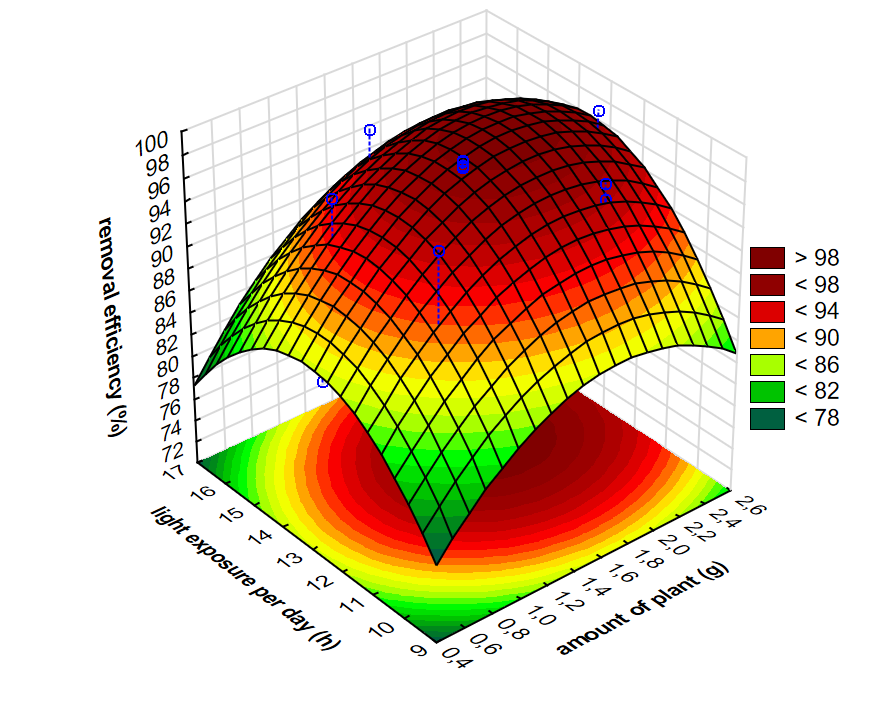 |
| --- |
| 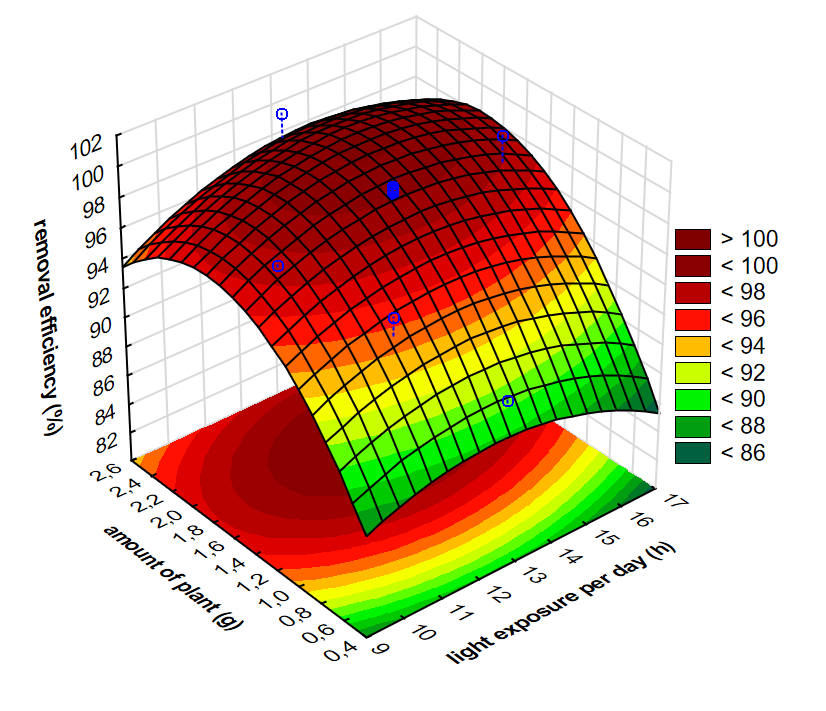 |
| 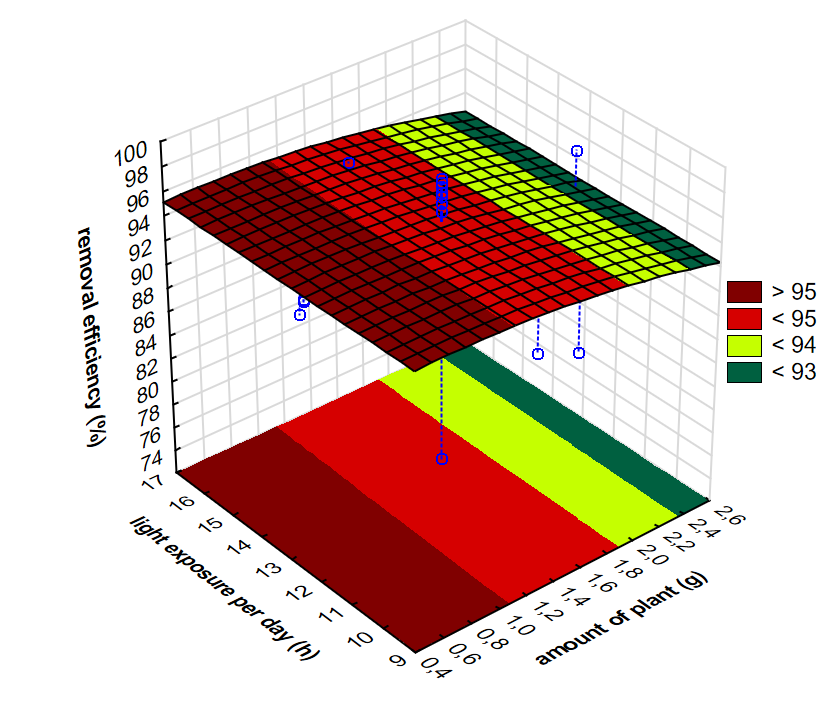 |

Figure S4. Effects of light exposure time, and mass of plant on EDCs removal rate by *W*. *arrhiza*

Table S7. The rate constant (*k*) values (day^-1^) of CECs removal by *W. arrhiza*

|  | *k_1_* | *k_2_* | *k_3_* | *k_5_* | *k_7_* | *k_14_* | *k _0-7_* | *k _0-14_* |
| --- | --- | --- | --- | --- | --- | --- | --- | --- |
| *C* = 100 µg/L | | | | | | | | |
| DEET | 0.67 | 0.50 | 0.12 | 0.26 | 0.15 | 0.07 | 0.30 | 0.19 |
| TRC | 1.33 | 1.02 | 1.10 | 0.27 | 0.01 | 0.20 | 0.57 | 0.39 |
| BPA | 1.67 | 0.30 | 0.55 | 0.58 | 0.14 | 0.23 | 0.57 | 0.40 |
| DES | 0.59 | 1.33 | 0.02 | 0.68 | -0.05 | 0.53 | 0.46 | 0.49 |
| E1 | 1.12 | 0.65 | 0.43 | 0.66 | 0.11 | 0.15 | 0.53 | 0.34 |
| E2 | 1.44 | 0.62 | 0.44 | 0.11 | 0.36 | 0.14 | 0.49 | 0.32 |
| *C* = 500 µg/L | | | | | | | | |
| TRC | 1.20 | -* | 0.23 | 0.92 | -0.14 | - | 0.46 | - |
| BPA | 1.05 | - | -0.04 | 0.78 | 0.14 | - | 0.40 | - |
| DES | 1.43 | - | 0.35 | 0.44 | 0.00 | - | 0.43 | - |
| E1 | 0.97 | - | 0.15 | 0.63 | 0.35 | - | 0.46 | - |

* not determined

Table S8. The rate constant (*k*) values (day^-1^) of CECs removal by *L. minor*

|  | *k_1_* | *k_2_* | *k_3_* | *k_5_* | *k_7_* | *k_14_* | *k _0-7_* | *k _0-14_* |
| --- | --- | --- | --- | --- | --- | --- | --- | --- |
| *C* = 100 µg/L | | | | | | | | |
| DEET | 0.20 | 1.08 | 0.24 | 0.00 | 0.27 | 0.02 | 0.30 | 0.16 |
| TRC | 1.20 | 1.13 | 0.28 | 0.12 | 0.53 | 0.01 | 0.56 | 0.28 |
| BPA | 0.29 | 2.25 | -0.11 | 0.17 | 0.43 | 0.04 | 0.52 | 0.28 |
| DES | 0.47 | 2.84 | 0.07 | 1.76 | 0.00 | 0.00 | 0.99 | 0.49 |
| E1 | 0.48 | 1.92 | 0.17 | 0.18 | 0.30 | 0.06 | 0.50 | 0.28 |
| E2 | 0.46 | 1.24 | 0.31 | 0.24 | 0.39 | 0.00 | 0.47 | 0.23 |
| *C* = 500 µg/L | | | | | | | | |
| TRC | 0.84 | -* | 0.08 | 1.00 | -0.17 | - | 0.38 | - |
| BPA | 0.82 | - | 0.16 | 0.41 | 0.00 | - | 0.28 | - |
| DES | 0.13 | - | 0.61 | 0.31 | -0.07 | - | 0.26 | - |
| E1 | 0.25 | - | 0.24 | 0.69 | -0.08 | - | 0.28 | - |

* not determined

Table S9. The CECs half-life (*t_1/2_*, days) values in systems with *W. arrhiza* and *L. minor*

|  | *W. arrhiza* | | *L. minor* | |
| --- | --- | --- | --- | --- |
|  | *C* = 100 µg/L | *C* = 500 µg/L | *C* = 100 µg/L | *C* = 500 µg/L |
| DEET | 2.29 | - | 2.33 | - |
| TRC | 1.21 | 1.51 | 1.24 | 1.82 |
| BPA | 1.22 | 1.72 | 1.33 | 2.47 |
| DES | 1.52 | 1.62 | 0.70 | 2.65 |
| E1 | 1.30 | 1.51 | 1.38 | 2.47 |
| E2 | 1.41 | - | 1.48 | - |
